# Giant basal cell carcinoma of the scalp

**DOI:** 10.11604/pamj.2014.19.142.4619

**Published:** 2014-10-14

**Authors:** Monia Youssef, Hichem Belhadjali

**Affiliations:** 1 Dermatology Departement Fattouma Bourguiba, Hospital Monastir, Monastir, Tunisia

**Keywords:** Giant, basal cell carcinoma, scalp

## Image in medicine

A 64-year-old man, phototype V in Fitzpatrick scale, presented with a 5-year history of a slowly extending ulcer of the scalp. He had a past medical history of diabetes mellitus and ionizing radiation for tinea capitis in childhood. The physical examination revealed an oval-shaped ulcer measuring 7 cm in width x 5 cm in length, with a raised pigmented border; the center was alopecic, scattered by hemorrhagic, necrotic and crusted erosions. He had neither cervical enlarged lymph nodes nor visceromegaly. The histopathological study of the biopsy specimen confirmed the diagnosis of basal cell carcinoma. A combined cranial, thoracic, abdominal and pelvic computed tomography scan was normal. Particularly, there was no adjacent bone involvement. A surgical excision with reconstruction was undergone. No signs of dissemination or local recurrence have been detected after follow up of two years. We still observe on the 21^st^ century cases of giant basal cell carcinoma. In our case multiple factors could explain the size reached by the tumor: immunocompromisation (diabetes), ionizing radiation in childhood and mainly patient neglect. Ignorance about skin tumors and their prognosis, acceptance of a non painful, a slow growing lesion explain the delay of diagnosis and treatment. We have reported this case to increase awareness among dermatologists and general physicians about the importance of detecting this type of “quiet silent” tumor that if left could lead to dramatic complications. Final diagnosis: Giant basal cell carcinoma of the scalp.

**Figure 1:**
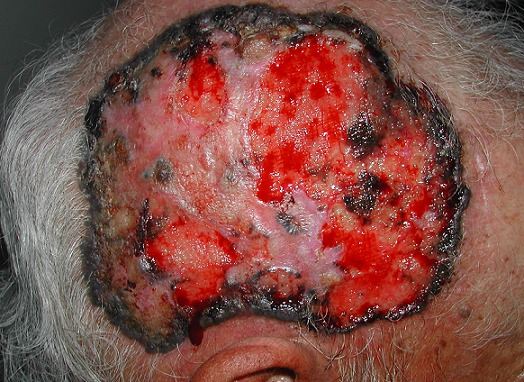
A large ulcer with raised pigmented border of the scalp

